# Serum fatty acid-binding protein 4 levels and responses of pancreatic islet β-cells and α-cells in patients with type 2 diabetes

**DOI:** 10.1186/s13098-021-00690-z

**Published:** 2021-06-26

**Authors:** Hong Wang, Jie Cao, Jian-bin Su, Xue-qin Wang, Xing Wang, Dong-mei Zhang, Xiao-hua Wang

**Affiliations:** 1 Department of Endocrinology, Affiliated Hospital 2 of Nantong University, and First People’s Hospital of Nantong City, No. 6, Haierxiang North Road, Nantong, 226001 China; 2 Medical Research Center, Affiliated Hospital 2 of Nantong University, and First People’s Hospital of Nantong City, No. 6, Haierxiang North Road, Nantong, 226001 China

**Keywords:** FABP4, β-cell function, α-cell function, Type 2 diabetes

## Abstract

**Background:**

Serum fatty acid-binding protein 4 (FABP4), as an intracellular lipid chaperone and adipokine, was reported to be related to the incidence of type 2 diabetes (T2D) and diabetic complications, but its association with pancreatic islet β-cell and α-cell functions has not been fully elucidated. So the present study was to investigate the serum FABP4 levels and responses of islet β-cells and α-cells in patients with T2D.

**Methods:**

115 patients with T2D and 89 healthy controls (HC), who received serum FABP4 levels test, were recruited to participate in this study. Moreover, 75-g oral glucose tolerance test (OGTT) was performed in T2D patients to evaluate islet β-cell and α-cell functions. Systemic insulin sensitivity and overall insulin secretion of islet β-cell function were assessed by Matsuda index using C peptide (ISI_M-cp_) and ratio of the area under the C peptide curve to the glucose curve (AUC_cp/glu_) during OGTT, respectively. Fasting glucagon (Gluca_0min_) and postchallenge glucagon assessed by the area under the glucagon curve (AUC_gluca_) were determined during OGTT to evaluate islet α-cell function. And other various clinical variables were also measured in all participants. Skewed variables were natural log-transformed (ln), such as lnFABP4.

**Results:**

The serum FABP4 levels in T2D patients were significantly higher than those in HC (*p* < 0.05). And after partially adjusting for fasting plasma glucose, serum lnFABP4 levels were negatively correlated with lnISI_M-cp_ (*r* =  − 0.332, *p* < 0.001) and positively correlated with lnAUC_cp/glu_ (*r* = 0.324, *p* < 0.001), lnGluca_0min_ (*r* = 0.200, *p* = 0.040) and lnAUC_gluca_ (*r* = 0.311, *p* < 0.001), respectively, in patients with T2D. Furthermore, when multiple linear regression analyses were applied to adjust for other various clinical variables, serum lnFABP4 levels were found to remain associated with lnISI_M-cp_ (*β* =  − 0.296, *t* =  − 2.900, *p* = 0.005), lnAUC_cp/glu_ (*β* = 0.223, *t* = 2.038, *p* = 0.046), lnGluca_0min_ (*β* = 0.272, *t* = 2.330, *p* = 0.024) and lnAUC_gluca_ (*β* = 0.341, *t* = 3.065, *p* = 0.004), respectively.

**Conclusion:**

Increased serum FABP4 levels were closely associated with blunted insulin sensitivity, increased insulin secretion, and elevated fasting and postchallenge glucagon levels in patients with T2D.

**Supplementary Information:**

The online version contains supplementary material available at 10.1186/s13098-021-00690-z.

## Background

Type 2 diabetes (T2D) has been known as ‘bi-hormonal disorder’, with pancreatic islet β-cell dysfunction responsible for the incidence and progression of T2D [1] and aberrant α-cell secretion responsible for the exacerbation of glycemic disorders [2, 3]. The core pathogenesis of T2D involves islet β-cell and α-cell dysfunctions. Pancreatic islet β-cell dysfunction of T2D is presented with blunted insulin sensitivity and/or abnormal insulin secretion, and islet α-cell dysfunction is characterized by elevated fasting glucagon and impaired repression of glucagon secretion after oral glucose load [4–7]. At present, ongoing research efforts worldwide are trying to screen risk factors for pancreatic islet β-cell and α-cell dysfunctions, which can help guide development of appropriate therapeutic regimens to improve these dysfunctions.

Fatty acid-binding protein 4 (FABP4), as a member of the FABPs family, is mainly expressed in adipocytes and macrophages. Circulating FABP4 mainly secreted from adipocytes and macrophages seems to not only function as lipid chaperone for the fatty acids transportation, storage and metabolism in target organs, but also serves as a bioactive adipokine in several target cells, including adipocytes, macrophages, endothelial cells, etc [8].

Under pathological conditions, excessive or ectopic expression of FABP4 was demonstrated to play an important role in the incidence and progression of metabolic diseases, such as metabolic syndrome and T2D. Increased levels of serum FABP4 have been found to be associated with increased risk of T2D in several follow-up studies [9, 10]. Moreover, in recent studies, increased serum FABP4 levels were also shown to predict the occurrence of micro- and macro-vascular complications in T2D, such as diabetic retinopathy (DR), diabetic nephropathy (DN), coronary artery disease (CAD) and acute ischemic stroke (AIS) [11–14]. Therefore, we hypothesized that increased serum FABP4 levels may get involved with the core pathogenesis of T2D. Some previous studies had shown that serum FABP4 levels were negatively correlated with the glucose-disposal rate in T2D (*n* = 18) [15] and positively correlated with glucose-stimulated insulin secretion in non-diabetic humans (*n* = 17) [16]. However, so far, there are few studies that have comprehensively analyzed the associations of serum FABP4 levels with overall pancreatic islet β-cell and α-cell functions in patients with T2D.

Therefore, the present study was conducted to systemically investigate the serum FABP4 levels and responses of β-cells and α-cells in patients with T2D.

## Methods

### Study design

We posted an announcement at the Department of Endocrinology and Health Examination Center of Affiliated Hospital 2 of Nantong University to recruit patients with T2D and healthy controls (HC), respectively, for this study from April 2019 to September 2020. The inclusion criteria for patients with T2D were as follows: (1) aged from 20 to 75 years, (2) diagnosis of T2D based on the statement published by the American Diabetes Association in 2015 [17]. The exclusion criteria for patients with T2D were as follows: (1) type 1 diabetes and other types of diabetes, (2) hyperglycemic crisis, (3) severe cardiac, hepatic or renal disease, (4) previous malignant tumors, (5) thyroid dysfunction, (6) recent use of glucocorticoids or immunosuppressive agents, (7) systemic autoimmune diseases. The inclusion criteria for HC participants were as follows: (1) aged from 20 to 75 years, (2) fasting plasma glucose (FPG) in the range of 3.9 and 6.1 mmol/L, (3) negative medical histories, (4) normal physical examinations, (5) normal hematological indices, (6) normal lipid profiles, (7) normal liver and renal function tests, (8) normal resting electrocardiograms.

Finally, 115 T2D patients and 89 HC were recruited for the present study. The study was approved by the Ethics Committee of Affiliated Hospital 2 of Nantong University and followed the principles of the Declaration of Helsinki. Furthermore, each participant provided an informed consent when they were enrolled.

### Clinical variables collection

Demographic information was collected, such as age, gender, height, weight, waist circumference (WC) and blood pressure. Waist circumference (WC) was measured at the level of umbilicus. Body mass index (BMI) was determined as weight divided to height squared (kg/m^2^). Systolic blood pressure (SBP) and diastolic blood pressure (DBP) were measured by an automatic blood pressure monitor after at least 30 min of rest. In patients with T2D, glucose-lowering therapies were also collected, such as lifestyle alone, insulin treatments, insulin secretagogues, metformin, pioglitazone, α-glucosidase inhibitors (AGIs), glucagon-like peptide-1 receptor agonists (GLP-1RAs) and dipeptidyl peptidase-4 inhibitors (DPP-4Is).

Peripheral fasting blood samples were collected after fasting for at least 8 h for the measurement of FPG, high-density lipoprotein cholesterol (HDLC), low-density lipoprotein cholesterol (LDLC), triglycerides (TG), total cholesterol (TC), serum creatinine (SCr) and uric acid (UA). Moreover, patients with T2D were received glycosylated hemoglobin (HbA1c) test. Renal function was assessed by estimated glomerular filtration rate (eGFR), which was calculated using the equation from Modification of Diet in Renal Disease (MDRD) Study [18]. Additionally, fasting blood samples were also drawn and centrifuged at 3000 g for 10 min, immediately divided into aliquots, and frozen at − 80 °C until centralized analysis for FABP4 levels. Serum FABP4 levels were measured by Solid Phase Sandwich ELISA at Medical Research Center (Human FABP4 Quantikine ELISA Kit; R&D Systems).

### Assessment of pancreatic islet β-cell and α-cell functions

All patients with T2D also underwent a 75-g oral glucose tolerance test (OGTT) for assessment of pancreatic islet α-cell and β-cell functions. Venous blood samples were collected at fasting (0 min) and at 30, 60, 120, and 180 min after glucose loading for the synchronous determination of serum glucose, C-peptide and glucagon levels. We used C-peptide instead of insulin in the β-cell function indices to eliminate cross-contamination of exogenous insulin with detection reagents. Systemic insulin sensitivity and overall insulin secretion of islet β-cell function were assessed by Matsuda index using C peptide (ISI_M-cp_) [19] and ratio of the area under the C peptide curve to the glucose curve (AUC_cp/glu_) during OGTT, respectively. Fasting glucagon (Gluca_0min_) and postchallenge glucagon assessed by the area under the glucagon curve (AUC_gluca_) were determined during OGTT to evaluate islet α-cell function.

### Statistical analysis

Clinical variables are displayed for patients with T2D and healthy controls (HC), and are expressed as mean ± standard deviation for normally distributed data, as the median(25% and 75% interquartile range) for skewed continuous data and as the frequency(percentage) for categorical data. To compare the differences in clinical variables between the two groups, Student’s t-tests, Mann–Whitney U tests or chi-square tests were used as appropriate, and corresponding test statistics (*t*, *Z* and *χ*^*2*^ values) and *p* values were also provided. If the data was non-normally distributed, a natural logarithm transformation (ln) was applied to achieve a normal distribution for further correlation and regression analysis, such as lnFABP4, lnISI_M-cp_, lnAUC_cp/glu_, lnGluca_0min_ and lnAUC_gluca_.

Pearson’s bivariate correlation analyses were applied to explore the correlations of lnFABP4 with lnISI_M-cp_, lnAUC_cp/glu_, lnGluca_0min_, lnAUC_gluca_ and other metabolic variables. Considering that FPG may have an effect on the FABP4 levels, the correlations of lnFABP4 with lnISI_M-cp_, lnAUC_cp/glu_, lnGluca_0min_ and lnAUC_gluca_ were adjusted for FPG by partial correlation analyses.

Furthermore, multiple linear regression analyses were applied to adjust for other various clinical variables in order to explore the independent effects of lnFABP4 on lnISI_M-cp_, lnAUC_cp/glu_, lnGluca_0min_ and lnAUC_gluca_. The initial model 0 was unadjusted; model 1 was adjusted for age, gender, BMI, WC, diabetic duration, SBP, DBP, TG, TC, HDLC, LDLC, eGFR, UA, FPG, HbA1c and glucose-lowering therapies. SPSS for Windows, standard version 23.0 (IBM Co., Armonk, NY, USA) was used to input and analyse the clinical variables, and *p* value < 0.05 could be considered statistically significant.

## Results

### Characteristics of HC and patients with T2D

**Fig. 1:**
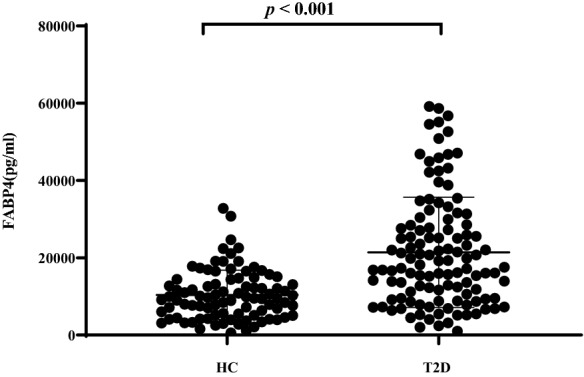
Levels of serum FABP4 in HC and patients with T2D

Characterisitics of clinical variables in HC and patients with T2D were shown in Table 1. Serum FABP4 levels of HC and patients with T2D were 9498.46 (5168.19−14406.19) pg/ml and 17535.96 (9513.62−28436.53) pg/ml, respectively. And serum FABP4 levels were obviously higher in patients with T2D than in HC (*p* < 0.001), which was also graphically displayed in Fig. 1. Moreover, serum FABP4 levels were found to be higher in female with T2D than in male with T2D (*p* = 0.012), with respective levels of 22677.00 (14964.61−34450.90) pg/ml and 16003.61 (8255.95−25362.95) pg/ml. Additionally, T2D patients had a higher male ratio, SBP, BMI and FPG, and a lower HDLC levels when compared to HC (all *p* < 0.05). However, there were no differences in age, DBP, TG, TC, LDLC, eGFR and serum UA between the HC and patients with T2D.

**Table 1:** Characterisitics of clinical variables in HC and patients with T2D

Variables	HC	T2D	*t*/*Z* /*χ*^*2*^ value	*p* value
n	89	115	−	−
Female, n (%)	47 (52.8)	42 (36.5)	5.412	0.020
Age (years)	55.06 ± 8.82	55.32 ± 11.42	0.128	0.856
BMI (kg/m^2^)	23.00 ± 2.42	24.92 ± 3.81	4.375	0.001
WC (cm)	−	90.80 ± 9.41	−	−
SBP (mmHg)	121.53 ± 10.98	132.84 ± 16.39	5.890	< 0.001
DBP (mmHg)	78.17 ± 7.53	79.21 ± 10.70	0.823	0.412
Diabetic duration (year)	−	8 (2−10)	−	−
Glucose-lowering therapies				
Lifestyle alone, n (%)	−	32 (27.8)	−	−
Insulin treatments, n(%)	−	35 (30.4)	−	−
Insulin secretagogues, n (%)	−	39 (33.9)	−	−
Metformin, n (%)	−	53 (46.1)	−	−
Pioglitazone, n (%)	−	10 (8.7)	−	−
AGIs, n (%)	−	13 (11.3)	−	−
GLP-1RAs, n (%)	−	1 (0.9)	−	−
DPP-4Is, n (%)	−	8 (7.0)	−	−
FPG (mmol/L)	5.18 ± 0.48	11.07 ± 3.58	17.426	< 0.001
TG (mmol/L)	1.33(1.01 − 1.86)	1.47 (0.96−2.84)	1.415	0.157
TC (mmol/L)	4.72 ± 0.72	4.73 ± 1.48	0.053	0.958
HDLC (mmol/L)	1.45 ± 0.32	1.17 ± 0.31	− 6.107	< 0.001
LDLC (mmol/L)	3.00 ± 0.62	2.91 ± 1.04	− 0.737	0.462
eGFR (ml/min/1.73 m^2^)	128.36(92.48−151.86)	121.81 (90.93−146.78)	− 3.427	0.078
Serum UA (umol/L)	295.90 ± 75.06	317.00 ± 115.21	1.532	0.127
HbA1c (%)	−	9.60 ± 2.07	−	−
FABP4 (pg/ml)	9498.46 (5168.19−14406.19)	17535.96 (9513.62−28436.53)	6.107	< 0.001

Student t-tests, Mann–Whitney U tests and Chi-square tests were applied to detect differences in their corresponding type of data, and corresponding test statistics (*t*, *Z* and *χ*^*2*^ values) and *p* values were also provided

### Correlations of FABP4 with clinical variables

**Fig. 2:**
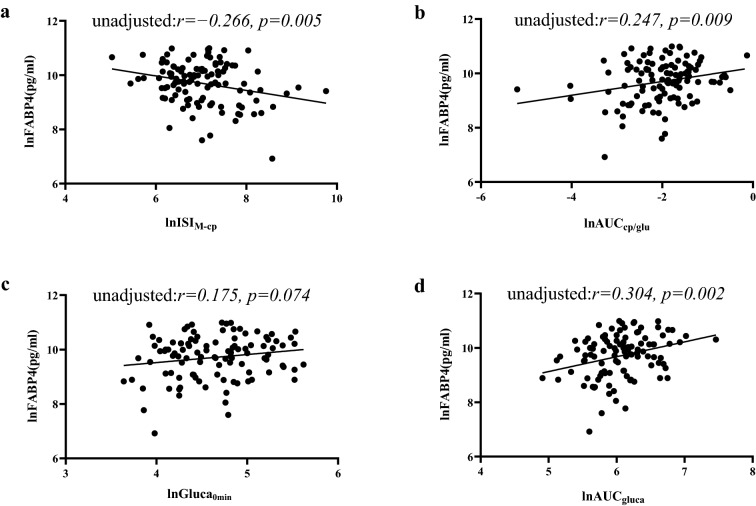
Relationships between serum FABP4 and islet β-cell and α-cell function indices in patients with T2D by univariate analysis (**a** ISI_M-cp_; **b** AUC_cp/glu_; **c** Gluca_0min_; **d** AUC_gluca_)

**Fig. 3:**
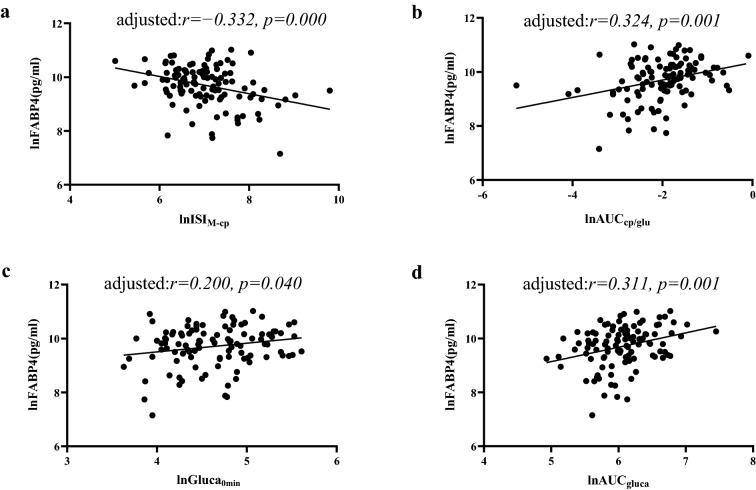
Relationships between serum FABP4 and islet β-cell and α-cell function indices in patients with T2D after adjusting for FPG (**a** ISI_M-cp_; **b** AUC_cp/glu_; **c** Gluca_0min_; **d** AUC_gluca_)

Pearson’s bivariate correlation analyses were applied to explore the correlations of lnFABP4 with metabolic variables (Table 2) and indices of islet β-cell function (lnISI_M-cp_ and lnAUC_cp/glu_) and α-cell function (lnGluca_0min_ and lnAUC_gluca_) (Table 3). Serum lnFABP4 was positively correlated with LDLC (*r* = 0.247, *p* = 0.020) in HC, and was positively correlated with FPG and UA in patients with T2D (*r* = 0.299 and 0.248, respectively, *p* < 0.05). Moreover, serum lnFABP4 levels were found to be related to lnISI_M-cp_, lnAUC_cp/glu_ and lnAUC_gluca_ (*r* =  − 0.266, 0.247 and 0.304, respectively, *p* < 0.05), but correlation of lnFABP4 with lnGluca_0min_ did not attain statistical significance (*r* = 0.175, *p* = 0.074). Furthermore, after adjusting for FPG by partial correlation analyses, correlations of lnFABP4 with lnISI_M-cp_, lnAUC_cp/glu_, lnGluca_0min_ and lnAUC_gluca_ became more evident (*r* =  − 0.332, 0.324, 0.200 and 0.311, respectively, *p* < 0.05). Scatter pots for these correlations were also displayed in Fig. 2 and Fig. 3.

**Table 2:** Correlations between the serum lnFABP4 and major metabolic variables in HC and patients with T2D

Variables	HC		T2D	
	*r*	*p* value	*r*	*p* value
FPG (mmol/L)	− 0.056	0.604	0.299	0.002
TG (mmol/L)	0.096	0.373	0.066	0.494
TC (mmol/L)	0.010	0.923	− 0.097	0.312
HDLC (mmol/L)	0.023	0.834	− 0.050	0.608
LDLC (mmol/L)	0.247	0.020	− 0.152	0.120
UA (umol/L)	0.022	0.840	0.248	0.011
eGFR (ml/min/1.73 m^2^)	− 0.067	0.232	− 0.127	0.196

**Table 3:** Correlations between the serum lnFABP4 and indices of β-cell and α-cell functions in patients with T2D

Variables	T2D	
	*r*	*p* value
lnCP_0min_ (ng/ml)	0.235	0.011
lnCP_30min_ (ng/ml)	0.264	0.005
lnCP_60min_ (ng/ml)	0.257	0.006
lnCP_120min_ (ng/ml)	0.226	0.016
lnCP_180min_ (ng/ml)	0.317	0.001
lnGluca_0min_ (pg/ml)	0.175	0.074
lnGluca_30min_ (pg/ml)	0.279	0.004
lnGluca_60min_ (pg/ml)	0.312	0.001
lnGluca_120min_ (pg/ml)	0.296	0.002
lnGluca_180min_ (pg/ml)	0.295	0.002
lnISI_M-cp_	− 0.266	0.005
lnAUC_cp/glu_	0.247	0.009
lnAUC_cp_	0.235	0.011
lnAUC_gluca_	0.304	0.002

Additionally, serum lnFABP4 levels were found to be correlated with C-peptide and glucagon levels at each point after glucose loading (Table 3). Serum lnFABP4 levels were correlated with lnISI_M-cp_ (*r* =  − 0.414, *p* = 0.018) in T2D patients without any glucose-lowering therapy (*n* = 32), and were correlated with lnISI_M-cp_ and lnAUC_cp/glu_ (*r* =  − 0.223 and 0.331, respectively, *p* < 0.05) in T2D patients with glucose-lowering therapies (*n* = 83) (Additional file 2: Figure S1). And the correlations of lnFABP4 with lnISI_M-cp_ and lnAUC_cp/glu_ remained significant in patients with T2D after adjusting for the glucose-lowering therapies (*r* =  − 0.265 and 0.235, respectively, *p* < 0.05) (Additional file 3: Figure S2). Serum lnFABP4 levels were also correlated with area under the C peptide curve from 0 to 30 min during OGTT (AUC_cp0–30 min_) in patients with T2D (*r* = 0.276, *p* = 0.003) (Additional file 4: Figure S3). When T2D patients were divided into subgroups according to diabetic duration, serum lnFABP4 levels were correlated with lnISI_M-cp_ (*r* =  − 0.349, *p* = 0.019) in T2D patients with diabetic duration ≤ 5 years, and were correlated with lnISI_M-cp_, lnAUC_cp/glu_ and lnAUC_gluca_ (*r* = − 0.245, 0.326 and 0.328, respectively, *p* < 0.05) in T2D patients with diabetic duration > 5 years (Additional file 1: Table S1).

### Multiple linear regression analyses exploring the impact of serum lnFABP4 levels on indices of islet β-cell and α-cell functions

Table 4 showed the associations of serum lnFABP4 levels with islet β-cell function (ISI_M-cp_, AUC_cp/glu_) and α-cell function (Gluca_0min_ and AUC_gluca_) via multiple linear regression analyses in patients with T2D. In the initial unadjusted model 0, serum lnFABP4 levels were significantly related to lnISI_M-cp_ (*β* =  − 0.266, *t* =  − 2.886, *p* = 0.005), lnAUC_cp/glu_ (*β* = 0.247, *t* = 2.662, *p* = 0.009) and lnAUC_gluca_ (*β* = 0.304, *t* = 3.240, *p* = 0.002), but association of lnFABP4 with lnGluca_0min_ did not attain statistical significance (*β* = 0.175, *t* = 1.807, *p* = 0.074). After adjusting for the other clinical variables in model 1, including age, gender, BMI, WC, diabetic duration, SBP, DBP, lipid profiles, eGFR, UA, FPG, HbA1c and glucose-lowering therapies, serum lnFABP4 levels remained independently associated with lnISI_M-cp_ (*β* =  − 0.296, *t* =  − 2.900, *p* = 0.005), lnAUC_cp/glu_ (*β* = 0.223, *t* = 2.038, *p* = 0.046) and lnAUC_gluca_ (*β* = 0.341, *t* = 3.065, *p* = 0.004), and became independently associated with lnGluca_0min_ (*β* = 0.272, *t* = 2.330, *p* = 0.024).

**Table 4:** Multivariate linear regression analyses displaying the impact of serum lnFABP4 levels on outcomes of lnISI_M-cp_, lnAUC_cp/glu_, lnGluca_0min_ and lnAUC_gluca_ in patients with T2D

Models	*β*	*t*	*p*	Adjusted *R*^*2*^ for model
lnISI_M-cp_				
Model 0	− 0.266	− 2.886	0.005	0.062
Model 1	− 0.296	− 2.900	0.005	0.391
lnAUC_cp/glu_				
Model 0	0.247	2.662	0.009	0.052
Model 1	0.223	2.308	0.046	0.351
lnGluca_0min_				
Model 0	0.175	1.807	0.074	0.021
Model 1	0.272	2.330	0.024	0.363
lnAUC_gluca_				
Model 0	0.304	3.240	0.002	0.084
Model 1	0.341	3.065	0.004	0.407

Model 0: unadjuseted

Model 1: adjusted for age, gender, BMI, WC, diabetic duration, SBP, DBP, lipid profiles, eGFR, UA, FPG, HbA1c and glucose-lowering therapies

## Discussion

In the present study, we compared the difference of serum FABP4 levels between the 115 patients with T2D and 89 healthy controls, and then analyzed the correlations of serum FABP4 levels with indices of pancreatic islet β-cell and α-cell functions. The main findings of our study were as follows: first, patients with T2D were presented with a higher levels of serum FABP4 when compared to healthy subjects; second, serum FABP4 levels were positively correlated with LDLC in healthy subjects and positively correlated with UA and FPG in patients with T2D; third, correlations of serum FABP4 levels with ISI_M-cp_, AUC_cp/glu_, Gluca_0min_ and AUC_gluca_ became more evident when partially adjusted for FPG; fourth, after adjusting for other various clinical variables, serum FABP4 levels were found to remain associated with ISI_M-cp_, AUC_cp/glu_, Gluca_0min_ and AUC_gluca_. Collectively, serum FABP4 levels were in close relation to the indices of pancreatic islet β-cell and α-cell functions.

### Serum FABP4 levels and metabolic diseases

Excessive expression or ectopic expression of FABP4 was proven to contribute to the multiple components of metabolic diseases and subsequent adverse outcomes, such as insulin resistance, dyslipidemia, obesity, metabolic syndrome, hypertension, type 2 diabetes, atherosclerosis and cardiovascular diseases [20–23]. In several previous studies, serum FABP4 levels were shown to be obviously increased in obesity and patients with T2D [22–25]. Kim et al [26] found that FABP4 was overexpressed in visceral tissues by proteomic analysis in patients with early T2D. And correlations of increased serum FABP4 levels with subcutaneous and visceral adipose depots evaluated by multi-slice computed tomography were almost the same [27]. Detection of serum FABP4 levels could be reflective of potential risk for metabolic diseases, such as obesity and T2D. FABP4 could enhance liver glucose production in vivo and in vitro, inhibit glucose oxidation and glycolysis, suppress uptake and utilization of glucose in muscles and liver [28], ultimately, lead to an increase in circulating glucose levels. Our study showed serum FABP4 levels were higher in the patients with T2D than in the healthy controls, and were significantly positively correlated with FPG in these T2D patients. Our finding is consistent with the previous studies. Moreover, in our present study, serum FABP4 levels were found to be higher in female with T2D than in male with T2D. And a previous study by Ibarretxe et al [29] also showed that serum FABP4 levels were determined by gender, which is in agreement with our study. The gender-difference of serum FABP4 is possibly due to two-fold: on the one hand, females tend to have a higher amount of body fat than males since there is a close correlation between serum FABP4 levels and adiposity [30]; and on the other hand, androgen may partially account for the gender difference in serum FABP4 levels [31].

### Serum FABP4 levels and indices of pancreatic islet β-cell function

Since serum FABP4 levels are correlated well with features of metabolic syndrome and are served as a predictor for the development of T2D. It is highly likely that increased serum FABP4 levels are involved in the core pathogenesis of T2D. A pervious study by Niu et al [32] found that serum FABP4 levels were positively correlated with homeostasis model assessment of insulin resistance (HOMA-IR), an indicator of hepatic insulin resistance, in a community population. Nakamura et al [15] found that serum FABP4 levels were negatively correlated with glucose-disposal rate in a small sample of T2D patients. Due to glucose-disposal rate primarily reflecting the sensitivity of skeletal muscle to insulin [33], increased FABP4 levels were closely related to decreased insulin sensitivity of muscle. Moreover, Nakamura et al [15] also observed that serum FABP4 was positively correlated with insulin secretion at 120 min of meal tolerance test in their study. Additionally, in a study with a cohort of 17 non-diabetic humans with BMI 19 from 36 kg/m^2^, Wu et al [16] found serum FABP4 levels were positively correlated with response in glucose-stimulated insulin secretion independence of body fat. And our present study revealed that increased serum FABP4 levels were independently associated blunted insulin sensitivity assessed by ISI_M-cp_ and overall increased insulin secretion of β-cells assessed by AUC_cp/glu_ in patients with T2D. ISI_M-cp_ is index of systemic insulin sensitivity, and can effectively reflect the sensitivity of the liver and peripheral tissues (mainly muscle) to insulin [19]. After analysis in subgroups, the association of FABP4 with ISI_M-cp_ was persisted in subgroups with and without glucose-lowering therapies, or in subgroups with different diabetic duration; while the significant association of FABP4 with AUC_cp/glu_ was restricted in subgroup who received glucose-lowering therapies or in subgroup with long-term of diabetic duration (Additional file 2: Figure S1 and Additional file 1: Table S1). Collectively, our study provided further evidence for the independent association of serum FABP4 levels with indices of islet β-cell function in T2D in the real world clinical practice.

The possible mechanisms for increased serum FABP4 linking to blunted insulin sensitivity may be as follows: first, overexpression of FABP4 contributes to endoplasmic reticulum stress, inflammatory response and oxidative stress [34], which in turn may lead to insulin resistance; second, elevated serum FABP4 levels are accompanied visceral adiposity, including ectopic fat deposition in islets of the pancreas, which may induce glucose intolerance, insulin resistance and β-cell dysfunction [35, 36]. The mechanisms for increased serum FABP4 linking to insulin secretion are also not well known, but some underlying mechanisms have been indirectly suggested. Serum FABP4 levels may result in insulin resistance, which in turn may lead to a compensatory increase in insulin secretion trying to maintain glucose homeostasis [37]. Additionally, in the basic studies, FABP4-deficient mice was found to have a reduction in insulin secretion [38, 39], which indicated that FABP4 participated in the insulin secretion; and FABP4 could potentiate glucose-stimulated insulin secretion in the presence of linoleic acid in vitro and in vivo. Moreover, neither FABP4 nor linoleic acid alone enhanced insulin secretion. Therefore, FABP4 and fatty acid have synergistic effect on β-cells to promote insulin secretion [16].

### Serum FABP4 levels and indices of pancreatic islet α-cell function

Our present study may be the first to analyze the relationship between serum FABP4 levels and indices of pancreatic α-cell function, and we found that increased serum FABP4 levels were associated with elevated fasting and postchallenge glucagon levels (Gluca_0min_ and AUC_gluca_) during OGTT in patients with T2D. In a previous study with 106 patients with T2D, Demant et al [40] identified that fasting glucagon levels were influenced by visceral fat deposition, and visceral fat deposition was paralleled by increased serum FABP4 levels. Moreover, hepatic insulin resistance, assessed by HOMA-IR, may lead to fasting hyperglucagonaemia [41], and HOMA-IR was also paralleled by increased serum FABP4 levels [32]. Additionally, palmitate can stimulate secretion of glucagon in isolated human islets via free fatty acid receptor at fasting glucose levels [42], and postprandial lipemia increased plasma glucagon concentrations in humans. Under a pathological condition such as obesity-induced oxidative stress, fatty acid (palmitate) would have relatively high affinity for FABP4, which would facilitate glucagon secretion from α-cells [8]. Furthermore, in pancreatic islets, β-cells and other cells (such as α-, δ- and PP-cells) would lose their identities under some certain conditions, such as glucolipotoxicity [43–45]. In a basic study with mouse, pancreatic islet β-cells were found to be transdifferentiated into α-cells when the β-cell-specific FoxO1 was inhibited under the condition of the glucotoxicity [45, 46]. In human studies with T2D, islet β-cell dysfunction was partly caused by transdifferentiation of β-cells into α-cells under glucolipotoxicity [44, 47]. FABP4 regulated the fatty acids, and may participate in the progress of transdifferentiation of β-cells into α-cells under lipotoxicity. Collectively, our study and the indirect evidence of previous studies showed increased serum FABP4 levels may account for the elevated fasting and postchallenge glucagon.

## Limitations

Our study has certain limitations. First of all, this study is a cross-sectional study that analyzed the correlations between serum FABP4 levels and pancreatic β-cell and α-cell functions. It is difficult to determine the causality of serum FABP4 levels and indices of islet β-cell and α-cell functions, so follow-up studies or basic researches are needed. Second, although relationship serum FABP4 levels and indices of islet β-cell and α-cell functions remain significant after adjusting for the glucose-lowering therapies, some antidiabetic drugs such as DPP-4Is may have impact on the levels of serum FABP4 [48]. Third, the golden standard for evaluating pancreatic β-cell functions is the glucose clamp test and the derivative indicators calculated from OGTT may not be able to replace assessments of insulin sensitivity and insulin secretion of β-cells. However, the glucose clamp test is too complicated and difficult to be applied in the clinical practice. Finally, the recruited populations of this study were Chinese and came from the same hospital, so our findings may not be extrapolated to other populations.

## Conclusions

Increased serum FABP4 levels were closely associated with blunted insulin sensitivity, increased insulin secretion, and elevated fasting and postchallenge glucagon levels in patients with T2D, which implied that excessive expression of FABP4 may get involved in the pancreatic islet β-cell and α-cell dysfunctions.

## Supplementary Information

**Additional file1:** **Table S1. **The relationships between serum lnFABP4 and indices of islet β-cell and α-cell functions in T2D patients with different diabetic duration.

**Additional file2:** **Figure S1. **The relationships between serum FABP4 and islet β-cell function indices in two subgroups of T2D patients without any glucose-lowering therapy (n=32) (**a **and **b**) and with glucose-lowering therapies (n=83) (**c **and **d**)

**Additional file3:** **Figure S2. **The relationships between serum FABP4 and islet β-cell function indices in patients with T2D after adjusting for the glucose-lowering therapies.

**Additional file4:** **Figure S3. **The relationship between serum FABP4 and AUC_cp0-30min_ in patients with T2D.

## Data Availability

The current data are available to all interested researchers upon reasonable request. Requests for access to data should be made to the principal investigators of the study.

## Abbreviations

FABP4: Fatty acid-binding protein 4
T2D: Type 2 diabetes
OGTT: Oral glucose tolerance test
ISI_M-cp_: Matsuda index using C peptide
AUC_cp/glu_: Ratio of the area under the C peptide curve to the glucose curve
Gluca_0min_: Fasting glucagon
AUC_gluca_: Area under the glucagon curve
DR: Diabetic retinopathy
DN: Diabetic nephropathy
CAD: Coronary artery disease
AIS: Acute ischemic stroke
HC: Healthy controls
FPG: Fasting plasma glucose
WC: Waist circumference
BMI: Body mass index
SBP: Systolic blood pressure
DBP: Diastolic blood pressure
AGIs: α-Glucosidase inhibitors
GLP-1RAs: Glucagon-like peptide-1 receptor agonists
DPP-4Is: Dipeptidyl peptidase-4 inhibitors
HDLC: High-density lipoprotein cholesterol
LDLC: Low-density lipoprotein cholesterol
TG: Triglycerides
TC: Total cholesterol
UA: Uric acid
HbA1c: Glycosylated hemoglobin
eGFR: Estimated glomerular filtration rate
HOMA-IR: Homeostasis model assessment of insulin resistance
