# Supplementary material for: Serum fatty acid-binding protein 4 levels and responses of pancreatic islet β-cells and α-cells in patients with type 2 diabetes
Source: Diabetol Metab Syndr. 2021 Jun 26;13:70. doi: 10.1186/s13098-021-00690-z (PMC8234651; doi:10.1186/s13098-021-00690-z)
Supplement: Supplementary file 1 — Additional file1: Table S1. The relationships between serum lnFABP4 and indices of islet β-cell and α-cell functions in T2D patients with different diabetic duration. [file 13098_2021_690_MOESM1_ESM.docx]

**Supplementary Table 1** The relationships between serum lnFABP4 and indices of islet β-cell and α-cell functions in T2D patients with different diabetic duration

| Variables | Diabetic duration ≤5 years  n=46 | |  | Diabetes durations >5 years  n=69 | |
| --- | --- | --- | --- | --- | --- |
|  | *r* | *p* |  | *r* | *p* |
| lnISI_M-cp_ | −0.349 | 0.019 |  | −0.245 | 0.047 |
| lnAUC_cp/glu_ | 0.062 | 0.686 |  | 0.326 | 0.008 |
| lnGluca_0min_ | 0.125 | 0.431 |  | 0.185 | 0.146 |
| lnAUC_gluca_ | 0.295 | 0.058 |  | 0.328 | 0.009 |
