# Supplementary material for: Serum fatty acid-binding protein 4 levels and responses of pancreatic islet β-cells and α-cells in patients with type 2 diabetes
Source: Diabetol Metab Syndr. 2021 Jun 26;13:70. doi: 10.1186/s13098-021-00690-z (PMC8234651; doi:10.1186/s13098-021-00690-z)
Supplement: Supplementary file 2 — Additional file2: Figure S1. The relationships between serum FABP4 and islet β-cell function indices in two subgroups of T2D patients without any glucose-lowering therapy (n=32) (a and b) and with glucose-lowering therapies (n=83) (c and d) [file 13098_2021_690_MOESM2_ESM.pdf]

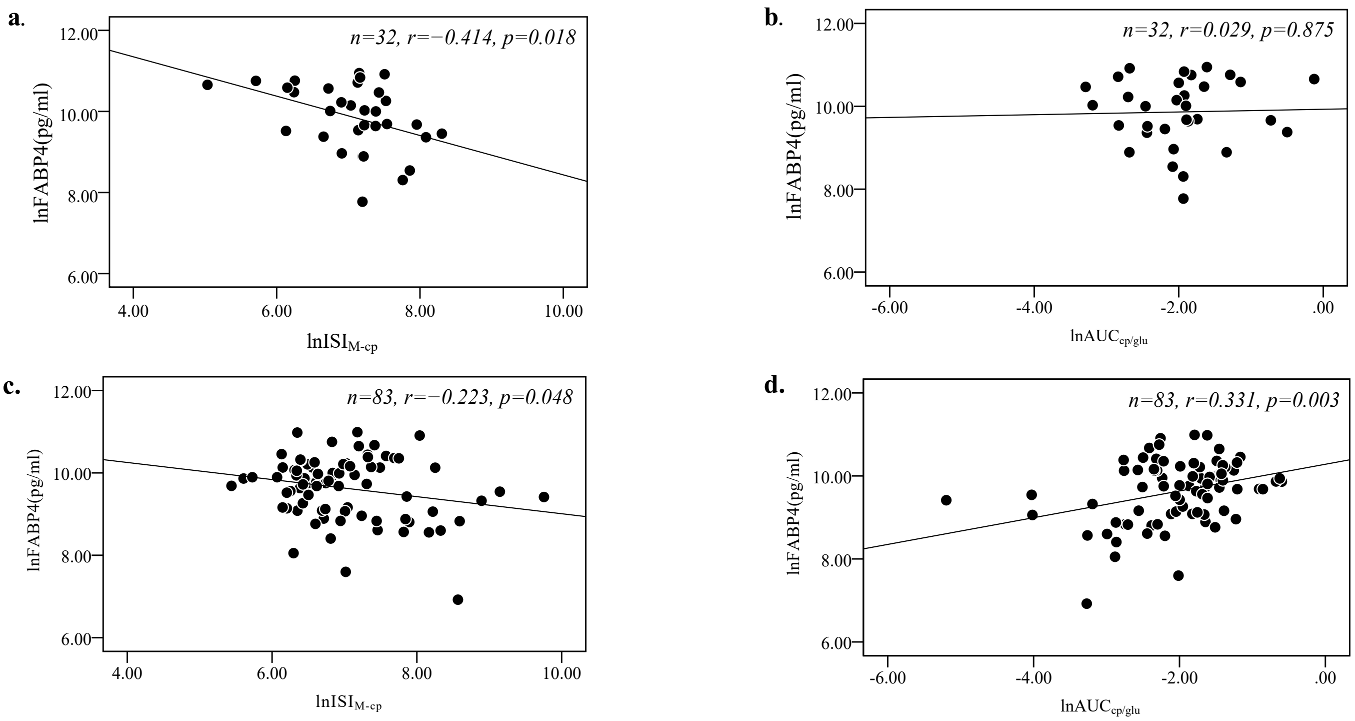

Supplementary Figure 1 The relationships between serum FABP4 and islet β-cell function indices in two subgroups of T2D patients without any glucose-lowering therapy (n=32) (**a** and **b**) and with glucose-lowering therapies (n=83) (**c** and **d**)
