# Supplementary material for: Serum fatty acid-binding protein 4 levels and responses of pancreatic islet β-cells and α-cells in patients with type 2 diabetes
Source: Diabetol Metab Syndr. 2021 Jun 26;13:70. doi: 10.1186/s13098-021-00690-z (PMC8234651; doi:10.1186/s13098-021-00690-z)
Supplement: Supplementary file 3 — Additional file3: Figure S2. The relationships between serum FABP4 and islet β-cell function indices in patients with T2D after adjusting for the glucose-lowering therapies. [file 13098_2021_690_MOESM3_ESM.pdf]

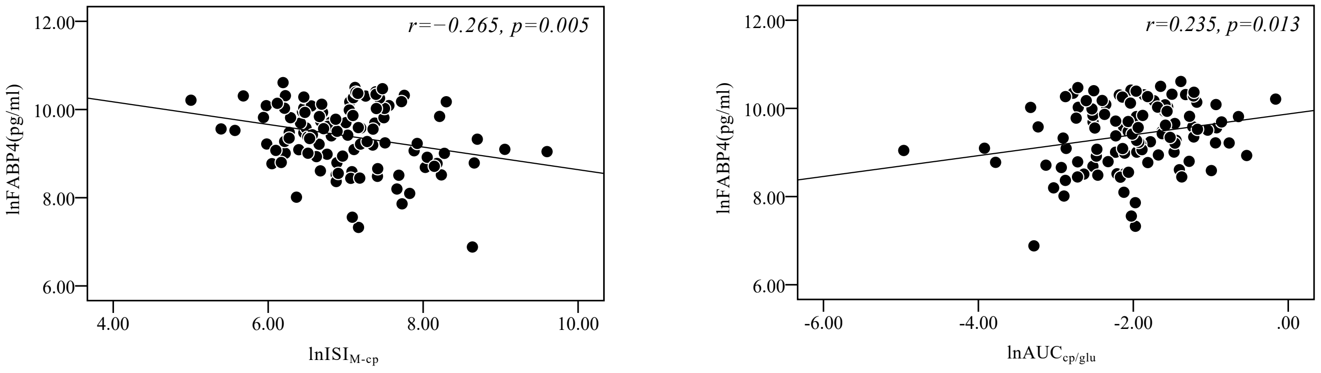

Supplementary Figure 2 The relationships between serum FABP4 and islet β-cell function indices in patients with T2D after adjusting for the glucose-lowering therapies
