# Supplementary figures and images for: Serum fatty acid-binding protein 4 levels and responses of pancreatic islet β-cells and α-cells in patients with type 2 diabetes
Source: Diabetol Metab Syndr. 2021 Jun 26;13:70. doi: 10.1186/s13098-021-00690-z (PMC8234651; doi:10.1186/s13098-021-00690-z)

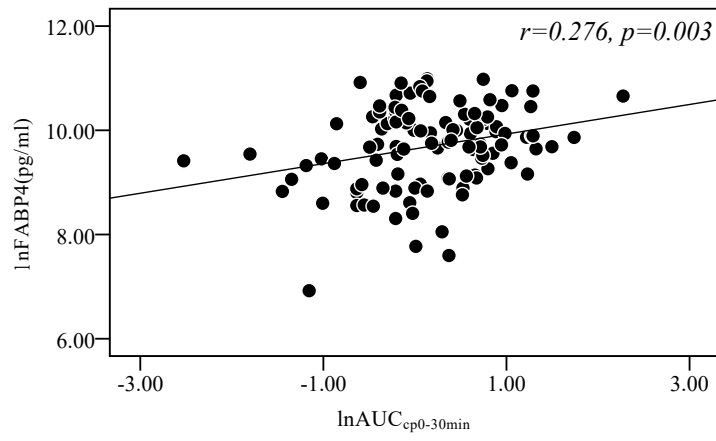

**Supplementary Figure 3** The relationship between serum FABP4 and  $AUC_{cp0-30min}$  in patients with T2D

Supplement: Supplementary file 4 — Additional file4: Figure S3. The relationship between serum FABP4 and AUCcp0-30min in patients with T2D. [file 13098_2021_690_MOESM4_ESM.pdf]
